# Development of a System for Additive Manufacturing of Ceramic Matrix Composite Structures Using Laser Technology

**DOI:** 10.3390/ma14123248

**Published:** 2021-06-12

**Authors:** Stefan Polenz, Willy Kunz, Benjamin Braun, Andrea Franke, Elena López, Frank Brückner, Christoph Leyens

**Affiliations:** 1 Fraunhofer Institute for Material and Beam Technology (IWS), 01277 Dresden, Germany; elena.lopez@iws.fraunhofer.de (E.L.); Frank.Brueckner@iws.fraunhofer.de (F.B.); christoph.leyens@iws.fraunhofer.de (C.L.); 2 Fraunhofer Institute for Ceramic Technologies and Systems (IKTS), 01277 Dresden, Germany; willy.kunz@ikts.fraunhofer.de; 3 Space Structures GmbH, 12435 Berlin, Germany; braun@spacestructures.de; 4 AXIAL Ingenieurgesellschaft für Maschinenbau mbH, 01445 Radebeul, Germany; a.franke@axial-dd.de; 5 Product and Production Development, Department of Engineering Sciences and Mathematics, Luleå University of Technology, 97187 Lulea, Sweden; 6 Institute of Materials Science, Technische Universität Dresden, 01069 Dresden, Germany

**Keywords:** ceramic matrix composite, additive manufacturing, laser technology, wavelength dependent absorption rate, integrating sphere measurement

## Abstract

Ceramic matrix composites (CMCs) are refractory ceramic materials with damage-tolerant behavior. Coming from the space industry, this class of materials is increasingly being used in other applications, such as automotive construction for high-performance brake discs, furnace technology, heat coatings for pipe systems and landing flaps on reusable rocket sections. In order to produce CMC faster and more cost-efficiently for the increasing demand, a new additive manufacturing process is being tested, which in the future should also be able to realize material joints and higher component wall thicknesses than conventional processes. The main features of the process are as follows. A ceramic fiber bundle is de-sized and infiltrated with ceramic suspension. The bundle infiltrated with matrix material is dried and then applied to a body form. During application, the matrix material is melted by laser radiation without damaging the fiber material. For the initial validation of the material system, samples are pressed and analyzed for their absorption properties using integrating sphere measurement. With the results, a suitable processing laser is selected, and initial melting tests of the matrix system are carried out. After the first validation of the process, a test system is set up, and the first test specimens are produced to determine the material parameters.

## 1. Introduction

The conventional production of fiber-reinforced ceramic components mainly takes place via polymer infiltration pyrolysis (PIP), melt infiltration process (MI), chemical vapor infiltration (CVI) or sintering processes [1,2,3,4,5,6,7,8,9]. Another method that is still being researched on a small laboratory scale is electrophoresis deposition [10]. In the following, these methods will be briefly described, and their advantages and disadvantages will be mentioned.

In the case of the PIP, a fiber preform is produced as the starting framework, e.g., made of carbon fibers. This is infiltrated with a matrix polymer in a temperature range between 150 and 300 °C, followed by the chemical crosslinking of the polymer. The resulting green body is referred to as carbon fiber-reinforced polymer (CFRP). By means of pyrolysis under inert conditions from 700 up to 1600 °C, a ceramic matrix composite (CMC) arises from the CFRP. Due to the volume shrinkage, which occurs during pyrolysis, the matrix is being pervaded by pores and cracks. By the repetition of the process, steps of pure filtration, crosslinking and pyrolysis several times, the resulting porosity can be reduced iteratively to a minimum. In the end, however, a low remaining porosity must be tolerated. The advantages and disadvantages of the PIP and other methods are summarized in Table 1.

The MI process is similar to the PIP process but enables the production of CMCs with a higher density. In this case, the PIP process is interrupted after the first pyrolysis. Instead of repeating the previous process steps, the remaining void is infiltrated with metal instead (for example, liquid silicon infiltration (LSI)). This process adaption enables the reduction of porosity. The infiltrated metal closes the pores. In order to avoid the damage of the fibers by metal, the infiltration process must be carried out as quickly as possible.

The third conventional method is the CVI. Again, the basis is a fiber braid stabilized near the final contour. The fiber preform is heated in an oven and then flushed by the process gas. The process gas reacts, and some of the reaction products deposit on the fibers and form the matrix of the CMC. The remaining reaction products are filtered out of the process chamber.

Another variant for the production of oxidic CMC is sintering for matrix generation. Here, the matrix material is produced from starting materials by high-temperature treatment. These matrix precursor materials have lower melting temperatures (1000 to 1200 °C) than the final matrix (about 1600 °C). This allows processing without damaging the oxidic fibers by too high temperatures during processing. The matrix material is milled to powders in the nanometer particle size range and then processed into suspensions. The slurries are introduced between the fibers and sintered at the sintering temperature with high volume shrinkage.

Another method for matrix generation of CMC that is not yet conventionally used is electrophoresis. This involves dispersing electrically charged ceramic matrix particles in a liquid. The matrix particles are then deposited on oppositely charged fiber surfaces and between the fibers (electrodes) in a direct current electric field. The spaces between the fibers are filled with matrix material until the capillaries between the fibers are closed so far that the charged particles can no longer infiltrate. Here too, pore space remains open. In the experiments presented below, a CMC is to be produced for the first time using laser technology (Coherent Diamond J-3 10.6 400 W OEM laser, Coherent, Palo Alto, CA, USA) and a computer-controlled laydown mechanism (Axial Ingenieurgesellschaft GmbH, Dresden, Germany). For this purpose, samples of the powder materials SiO_2_ and Y_2_O_3_ are first pressed and then melted by the laser. The SiC fiber material mixed into these samples as particles are given special consideration in the subsequent ceramography. It should not melt under the determined process parameters in order to avoid mixing with the matrix material and thus guarantee the typical CMC properties. The Y-Si-O matrix system was selected because it forms yttrium silicates in thermodynamic equilibrium, which have sufficient mechanical stability and excellent corrosion stability at both room and high temperatures [11,12,13]. Likewise, the coefficient of thermal expansion is close to that of SiC fibers, and there are no chemical interactions between the components [14,15].

## 2. Materials and Methods

A new process based on additive manufacturing methods, as published in the patent specification DE102015205595B3 [16], gives the opportunity to overcome long processing times and the dependency on furnace technology. A ceramic fiber bundle is to be continuously de-sized after unwinding from the fiber supply roll and then infiltrated with a ceramic slurry. Followed by a drying process, the infiltrated fiber bundle is positioned by a CNC-controlled depositing mechanism, and at the same time, the matrix material around the fibers is consolidated by means of laser treatment.

Compared to conventional methods, the new additive process offers various advantages (see Table 1). One of them is shaping. This is to take place via a quasi-continuous process step. As described in the patent, a fiber bundle is to be infiltrated with matrix material and then deposited. The aim is to melt the matrix powder during the depositing process and to compact it around the fibers without damaging them. Since the requirements for the matrix starting materials are lower than for sintering, they are currently cheaper to procure. The new method should make it possible to integrate metallic fastening elements and sensor technology during the process through shorter, local energy input in the manufacturing process. Joining is addressed as a further possible application of the process. The process can theoretically be scaled well, as the machining area of the unit is regarded as the limit and can be adapted to the necessary conditions.

The fiber material, SiC, was defined as the base point for the material system. The following conditions for the matrix system and the beam source to be used are derived from this:

- Comparatively high beam absorption of the matrix compared to the fiber material [17]
- Meltability of the matrix material
- Physical and chemical compatibility with SiC (coefficient of linear thermal expansion, chemical interactions, oxidation).

As can be seen from sources [13,14], yttrium disilicate (Y_2_Si_2_O_7_) seems to be suitable as a matrix material. However, the absorption values are not described. Based on other oxides [17], however, it seems reasonable to expect that equally high absorption values can be achieved here for CO_2_ lasers (λ = 10.6 μm). The higher absorption of the matrix materials should ensure that they melt before the fiber material with the lower absorption value is affected.

To confirm the assumption, the absorption values of the mentioned oxidic base materials (Y_2_O_3_, Grade C, Höganäs AB, Höganäs, Sweden; SiO_2,_ Zandosil 30, Heraeus, Hanau, Germany) were analyzed by integrating sphere measurements and compared with the literature values of SiC. The Varian Cary 5000 UV-VIS-NIR spectrometer (Agilent Technologies Inc., Santa Clara, CA, USA) was used to measure the absorbance values over a wavelength range of 0.3 to 15 µm. To analyze the absorption values, samples of powdery matrix material with different compositions were pressed. The method was as follows:

- Attrition milling in isopropyl alcohol for 4 h
- Uniaxial Pressing to 16 × 16 × 4 mm^3^;
- Cold isostatic pressing at 250 MPa
- Debinding at 600 °C for 1 h in air.

Table 2 gives an overview of the sample compositions tested.

**Figure 1:**
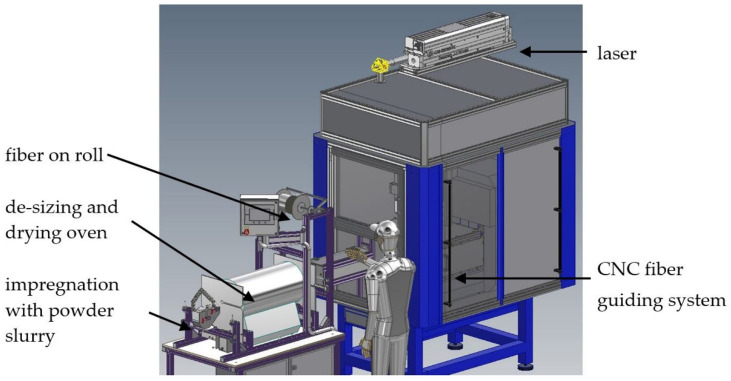
CAD model of the machine for additive manufacturing of CMC using laser technology.

Based on the analyzed oxidic materials, a matrix system will be selected. The corresponding phase diagram for the matrix material system consisting of SiO_2_ and Y_2_O_3_ can be found in source [18]. Following the material and laser selection, initial parameter tests are carried out for melting the pressed samples under variation of the laser power (P_L_) between 10 and 100 W, laser feed rate (v) between 200 and 16,000 mm/min and laser spot diameter (d) of 1 mm. The energy density (E) was considered as a comparative value. The samples were evaluated by means of ceramographic cross-sections, electron microscope and X-ray diffraction. The best parameter sets form the starting point for the first tests with the new additive manufacturing process described in Figure 1.

## 3. Results

**Figure 2:**
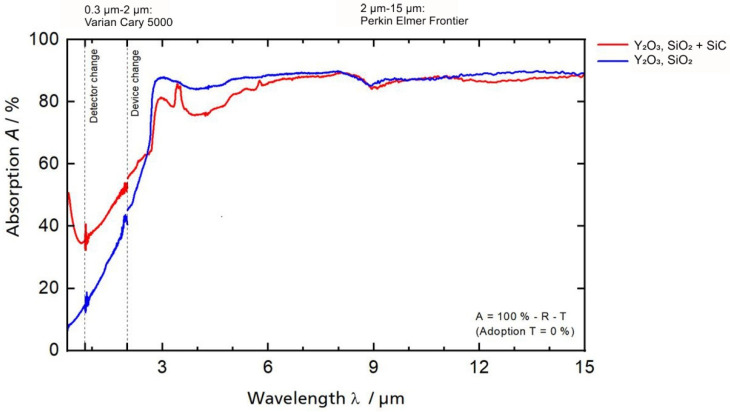
Absorption rate (A) of selected ceramic materials by different wavelengths (λ) using integrating sphere measurements.

Using the pressed samples prepared as described in Section 2, the integrating sphere measurement was carried out to determine the absorption values of different compositions of SiO_2_ and Y_2_O_3_. The results are shown in Figure 2.

From the results of the integrating sphere measurements, it can be seen that the selected oxide ceramic matrix materials SiO_2_ and Y_2_O_3_ show significantly higher absorption values (about 90%) at a comparatively high laser wavelength of 10.6 μm than the SiC fiber material with 66% [17]. Due to the proven difference in absorption values, the following tests are carried out with a mixture of SiO_2_ and Y_2_O_3_ in a stoichiometric ratio of 2:1. The composition is derived from the eutectic produced in this ratio and the associated low melting temperature (about 1775 °C [18].

**Figure 3:**
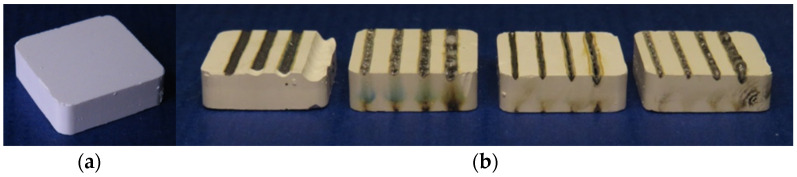
Pressed test specimens of Y_2_O_3_-SiO_2_ with 10 vol.% SiC, (**a**) before and (**b**) after first tests.

To investigate the effect of the molten bath on the fiber material, particulate silicon carbide (SiC; F1200, ESK-SIC GmbH, Frechen, Germany) with a volume fraction of 10% was added as a substitute to the matrix system. The powder mixtures were then mixed with organic binders, ground and processed into granules. This was formed into cuboids measuring 16 mm × 16 mm × 4 mm by cold isostatic pressing (see Figure 3). The organic material was then debound at temperatures above 500 °C.

**Figure 4:**
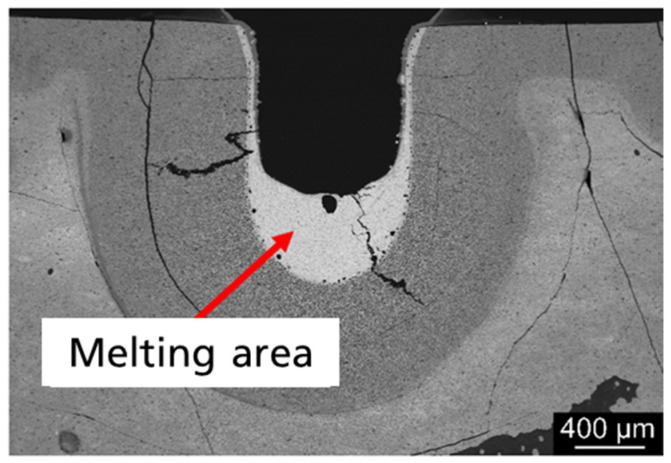
Cross-sections of Y_2_O_3_-SiO_2_ processed with λ = 10.6 µm.

The pressed samples were partially melted in the first experiments using a laser (Diamond J-3 10.6 400 W OEM Laser, Coherent, Palo Alto, CA, USA) of 10.6 µm wavelength. The investigation of the single-track tests showed a different reaction of the material to the laser parameter variation of laser power, feed rate and spot diameter. Figure 4 shows a cross-section of the track tests with λ = 10.6 µm for the material composite. The sample shows a homogeneous melt pool with a dense structure that has not solidified in thermodynamic equilibrium due to the extremely high cooling rate.

**Figure 5:**
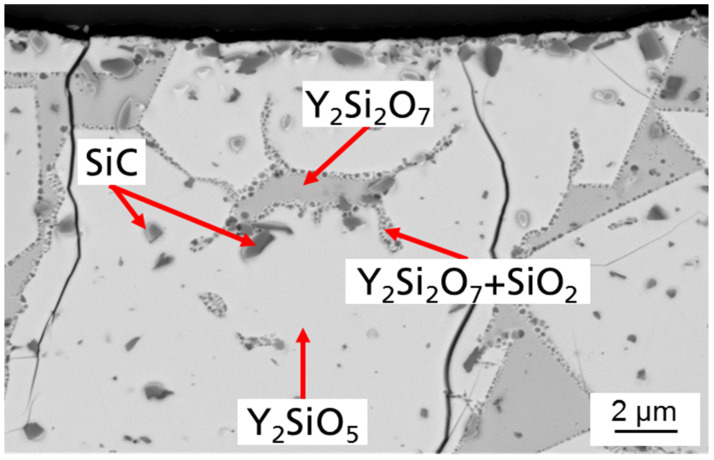
Cross-section of Y_2_O_3_-SiO_2_ sample with 10 vol.% SiC melted with λ = 10.6 µm.

A cross-section with higher enlargement of a press specimen with 10 vol.% SiC is shown in Figure 5. According to the phase diagram [18], it was to be expected that mainly yttrium disilicate with embedded SiC would form.

**Figure 6:**
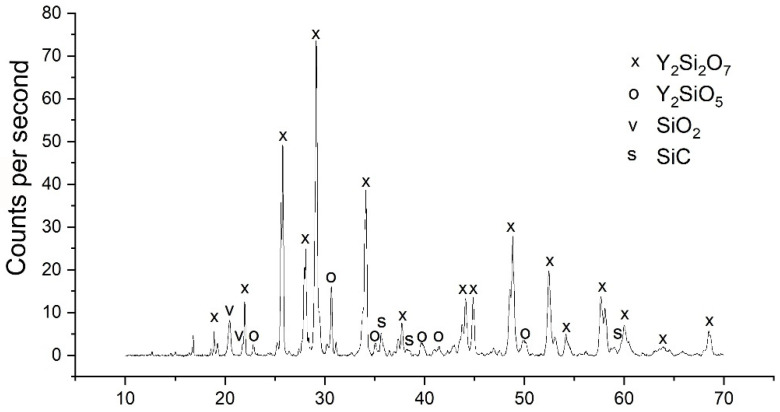
EDS (Energy dispersive spectroscopy) measured composition of melted Y_2_O_3_-SiO_2_ sample with 10 vol.% SiC.

Measuring the composition by EDS (Energy dispersive spectroscopy) (Figure 6), four different phases could be identified by the stoichiometric ratio of the elements: yttrium disilicate, yttrium monosilicate, silica and silicon carbide. This non-equilibrium state is not in accordance with the phase diagram but is caused by the rapid cooling of the melt. However, the evaporation of Si-O species during processing leads to a partial reduction in the yttrium disilicate with the formation of more yttrium monosilicate compared to the residual amount of silica. A phase of yttrium disilicate with silicon oxide is deposited on the edge of the yttrium disilicate crystals by segregation of the yttrium monosilicate.

**Figure 7:**
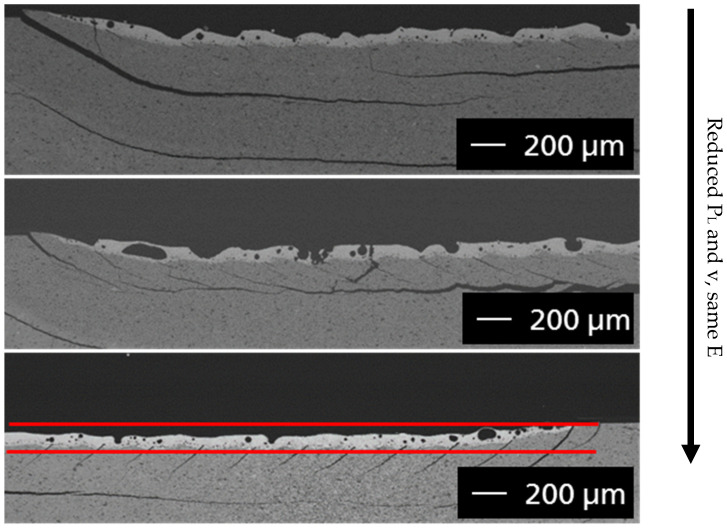
Cross-sections of surface melt tests from top to bottom, reduced laser power (P_L_) from 40 to 10 W, lower feed rate (v) from 3200 to 800 mm/min, same energy density (E) at 0.5 J/mm².

The cross-sections of the surface melt tests (Figure 7) show similar laser penetration depths of around 140 µm from top to bottom, with reduced laser power, lower feed rate and the same energy density. Viewed from the surface, the samples show no macroscopic defects (delamination, cracking, color change), even when repeatedly processing the same surface with the same parameters. This is essential for the planned welding process, as the layer-by-layer structure requires repeated laser exposure of a surface.

**Figure 8:**
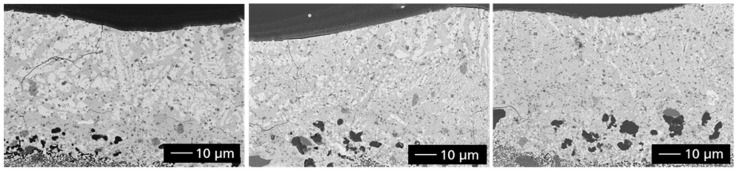
Structural variations from left to right—Concentration of Si-O and increased formation of yttrium disilicate (grey) due to reduced energy input and lower feed rate at the same energy density.

The characterization of the microstructure formation of the melting regions in Figure 7 was carried out by means of cross-sections and their analyses in the field emission scanning electron microscope (FESEM: NVision40 or Ultra55, Carl Zeiss AG, Oberkochen, Germany) (Figure 8) and X-ray diffraction (XRD: D8, Bruker AXS, Karlsruhe, Germany).

Depending on the power parameters of the laser, the chemical composition of the sample changes. Higher energy inputs lead to vaporization of Si-O and thus to a reduction of the yttrium disilicate with the formation of yttrium monosilicate. It is a benefit for the process to reduce the laser power and the feed rate at the same energy density. As the tests (Figure 8) show, this resulted in a higher amount of the disilicate phase in the cross-sections.

**Figure 9:**
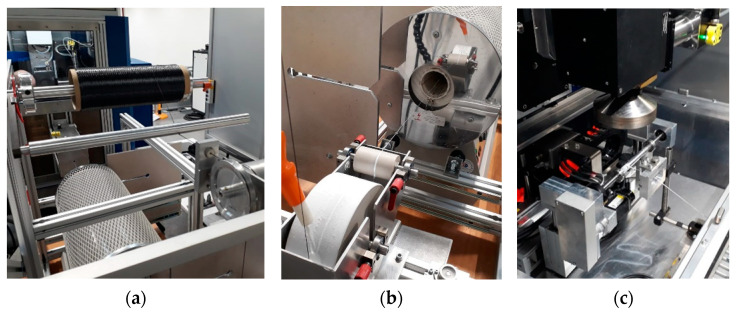
Details of the test set-up from left to right: (**a**) fiber bundle reservoir; (**b**) coating of the fiber bundle in the slurry with subsequent furnace drying; (**c**) fiber guide system.

Based on the results of the melting experiments on pressed powder tablets, the next step was to test the CMC production by laser technology on fiber composites consisting of Y_2_O_3_-SiO_2_ (1–2 mol) and the fiber material (Tyranno SA3, Ube Ind., Tokyo, Japan), as can be seen in the detailed images in Figure 9, the first samples were set up with the newly developed system.

**Figure 10:**
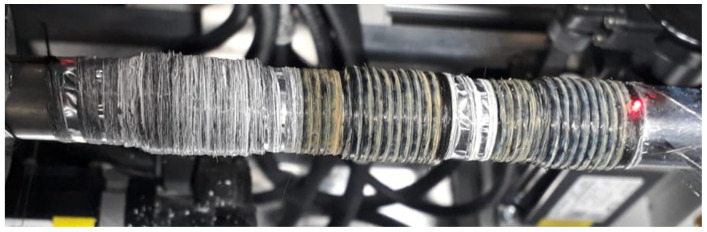
First Y_2_O_3_-SiO_2_ sample with SiC fiber, melted with λ = 10.6 µm, produced using the new system: without melting the matrix (left), with melted matrix (middle and right).

Following the functional tests of the system, the first samples were processed by laser, as can be seen in Figure 10.

**Figure 11:**
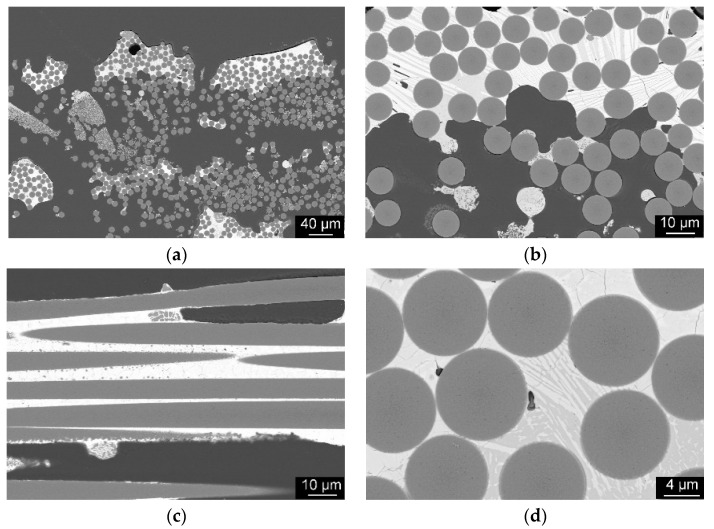
Cross-section Y_2_O_3_-SiO_2_ sample with SiC fiber, melted with λ = 10.6 µm, overview (**a**), higher magnification of the transition (**b**), cross-section along the fibers (**c**), area with embedded fibers in matrix material (**d**).

As can be seen in the cross-sections in Figure 11, it was confirmed that the fibers were not damaged by the laser, as expected. Damage only occurred in isolated cases where minimal matrix material was present. The transition areas between fiber and matrix correspond to the expectations of a CMC. In the images, the fibers appear to be embedded in the matrix. This is indicated by the grain structures of the matrix, which do not continue in the fiber, and a step in the cross-sections between fiber and matrix.

## 4. Conclusions

In summary, it can be said that in the experiments with the press specimens, the use of the wavelength of 10.6 µm made it possible to melt the matrix systems. The tests with SiC-fiber material showed that a homogeneous infiltration of the fiber bundles with matrix material during the winding process is possible. However, the amount of matrix material between the fibers of the fiber bundle still has to be increased. After the welding process, there is still insufficient matrix between the fibers. Due to the high wavelength of the CO_2_ lasers (λ = 10.6 µm), the laser power is predominantly absorbed close to the surface (100–200 µm). Thus, the melting process takes place close to the surface. In order to increase the penetration depth, the laser parameters are varied in further experiments (feed rate, laser power, laser relative movement). The fiber material (Tyranno SA3, Ube Industries, Tokyo, Japan) used was not damaged by the laser during processing. If the laser power was too high (over 100 W), mixing with the matrix material occurred on the fiber surface.

As an outlook for the processing technique of CMC using laser technology described in the paper, the following can be noted. The proportion of infiltrated matrix powder must be increased, by changing the binder system, or by increasing the penetration depth of the laser in the melting area under controlled energy density by varying the laser power, feed rate and scanning strategy. To prevent damage to the fibers, the laser power should be kept under 50 W and the feed rate over 4000 mm/min. The scanning strategy should be adapted so that the process zone is scanned several times. For example, oscillating, circular or rectangular scanning patterns of the laser beam could be used.

## Figures and Tables

**Table 1:** Advantages and disadvantages of different production methods for fiber-reinforced ceramic components [1,2,3,4,5,6,7,8,9,10,11].

Method	Advantages	Disadvantages
Polymer Infiltration Pyrolysis	- similarity in design and manufacturing with conventional CFRP standards and low tooling costs leads to cost-efficiency - possibility of in situ joining of pre-hardened parts in the autoclaving process	- high cost of ceramic precursors - very long process time - multiple repetitions of the infiltration, crosslinking and pyrolysis - high residual porosity and low interlamellar shear strength
Melt Infiltration	- processing time under 5 days - dense parts (porosity under 2%) - low cost - complex and near-net shapes may be fabricated - high electrical conductivity	- residual metal limits the application temperature - high process temperatures during infiltration - possible attack of fibers by metal
Chemical Vapor Infiltration	- high flexibility in the variety of shapes - in large ovens, many parts of different shapes can be infiltrated simultaneously - only one infiltration step	- very long process time - only small wall thicknesses (3–5 mm) processable - closed residual porosity with pore diameters up to 0.5 mm
Sintering	- use of conventional sintering technology - inexpensive - low processing temperatures at - 1000 to 1200 °C	- only for oxide matrix materials - porosity values around 20% - low operating temperatures around 1600 °C
Electrophoresis	- cold process - without fiber damage	- complex powder preparation and dispersion - limitation to thin component thicknesses - complex process technology - porosity
Laser-induced Fabrication of CMC	- simple processing - high flexibility in the variety of shapes - no size limitations - implementation of functional materials or components possible	- strength level unknown yet - sensitive fiber bundle during processing - elaborate fiber processing

**Table 2:** Composition of the pressed samples.

Sample	SiO_2_ (%)	Y_2_O_3_ (%)
1	100	0
2	0	100
3	66.6	33.3

## Data Availability

All the data is available within the manuscript.
